# A family with normal sperm motility carrying a sY86 deletion in AZFa region and partial deletion in AZFc region

**DOI:** 10.3389/fgene.2024.1519774

**Published:** 2025-01-09

**Authors:** Yuhong Zhao, Weiwei Zhi, Dongsheng Xiong, Ningjing Li, Xinrong Du, Jiuzhi Zeng, Guohui Zhang, Weixin Liu

**Affiliations:** ^1^ The Affiliated Women’s and Children’s Hospital of Chengdu Medical College, Sichuan Provincial Woman’s and Children’s Hospital, Chengdu, China; ^2^ Reproductive Medicine Center, Sichuan Provincial Woman’s and Children’s Hospital, Chengdu, China; ^3^ School of Medicine and life sciences, Chengdu University of Traditional Chinese Medicine, Chengdu, Sichuan, China

**Keywords:** azoospermia factor, sY86 deletion, whole-exome sequencing, Y-chromosome microdeletions, genetics

## Abstract

**Introduction:**

Usually, patients with sY84 or sY86 deficiency present with azoospermia, but recent studies have shown that some males with partial AZFa deletions, including sY84 or sY86, exhibit normal fertility. Here, we reported a rare case of AZF deletion in a family, where both father and son exhibited a deletion at the sY86 site in the AZFa region and a partial deletion in the AZFc region.

**Methods and Results:**

Detection was performed using classical multiplex polymerase chain reaction and the “Male AZF Full-region Detection” Panel, revealing specific deletions in AZFa: Yq11.21 (14,607,372–14,637,973), 30.6 kb; AZFc: Yq11.223–11.23 (25,848,831–27,120,665), 1.3 M for the father; and Yq11.223–11.23 (25,505,378–27,120,665), 1.6 M for the son. Notably, although the son’s sperm motility parameters showed no significant abnormalities, there was a history of failed pregnancies for twice, with sperm exhibiting a high rate of head defect.

**Discussion:**

Given the complexities of the reproductive phenotype following AZF region deletions, additional extended genetic testing is necessary when partial deletions in the AZF region are detected, thus providing more accurate predictions of the spermatogenesis in patient. This study provides valuable insights and guidance for clinical decision-making and the implementation of assisted reproductive technologies in such cases.

## 1 Introduction

With the changing lifestyle and increasing social pressures, the incidence of infertility has been steadily rising, currently affecting approximately 15% of couples of reproductive age, with male factor infertility accounting for around 50% (Ray et al., 2012; Agarwal et al., 2021). Male infertility is characterized by complex etiology and high phenotypic heterogeneity, which may stem from anatomical abnormalities, hormonal imbalances, reproductive system inflammations, and genetic factors. Currently, among the known genetic factors, abnormalities in chromosome structure and number, single gene defects, and Y-chromosome microdeletions (YCMs) contribute to approximately 15%–30% of male infertility cases (Hess and Renato de Franca, 2008; Leaver, 2016), Y chromosome microdeletions, in particular, constitute a genetic etiology for 15% of cases of severe oligozoospermia and azoospermia (Arumugam et al., 2021). Vogt et al. (1996) in 1996 delineated 76 discrete “microdeletion” sites within three subregions of Yq11 based on their roles in different stages of spermatogenesis in azoospermic males, categorizing them functionally as AZFa, AZFb, and AZFc regions, each harboring genes associated with male spermatogenesis impairment. Additionally, Kent-First et al. (1999) later discovered AZFd as a distinct gene structure located between AZFb and AZFc.

The detection rate of YCMs in infertile males exhibits significant geographic and racial disparities, with the rate of AZF deletions being 24% in Iran, 12% in the United States, and less than 2% in Germany and Austria (Cioppi et al., 2021). A study by Haiyang Yu et al. (2023) identified AZF deletions in 9% of 1,338 Chinese males diagnosed with azoospermia or severe oligozoospermia, with AZFc deletions accounting for 6%, and AZFa deletions approximately 0.8%. The AZF region on the Y chromosome encompasses several critical genes for spermatogenesis, and microdeletions in different regions may lead to oligozoospermia or azoospermia by affecting gene expression and function. Microdeletions in the AZFa region result in Sertoli cell-only syndrome (SCOS), clinically characterized by testicular atrophy and azoospermia (Liu et al., 2017). As the AZFa region harbors genes essential for spermatogenesis, its deletion implies the inability to obtain sperm even with procedures such as microdissection testicular sperm extraction. Deletions encompassing both AZFb and AZFc lead to either Sertoli cell-only syndrome or spermatogenic arrest, with affected individuals commonly presenting with azoospermia (Mahadevaiah et al., 1998; Yan et al., 2017). AZFc deletions constitute the most common type of AZF microdeletion, accounting for approximately 60% of Y chromosome microdeletions. In recent years, researchers have focused on “partial deletions” within the AZFc region due to its high phenotypic heterogeneity, manifesting as varying degrees of spermatogenic dysfunction: oligozoospermia and azoospermia (Kühnert et al., 2004; Kuo et al., 2004; Stahl et al., 2010). However, due to the possibility of producing normal sperm, individuals with AZFc deletions may represent the only subset of YCMs patients capable of siring biological offspring.

The European Academy of Andrology (EAA) and the European Molecular Genetics Quality Network (EMQN) recommend sY84 and sY86 as preferred sequence-tagged site (STS) for assessing AZFa deletions because their absence highly indicates complete AZFa deletion (Krausz et al., 2014). STS refers to short, single-copy DNA sequences with precise genomic locations that can be detected through polymerase chain reaction (PCR) (Olson et al., 1989), serving as landmarks in the human genome to determine the orientation of DNA and the relative position of specific sequences. In studies of the AZF region, STS are utilized as loci for detecting microdeletions. By examining these loci through PCR, we can ascertain the status of microdeletions in the AZF region of the Y chromosome, which is of great significance for diagnosing male infertility. However, recent studies have shown that a minority of males with partial deletions in the AZFa region, including those involving sY84 or sY86, exhibit normal spermatogenesis and fertility (Alksere et al., 2019; Jia et al., 2020; Tang et al., 2020). In our study, a father-son pair from the same lineage exhibited a deletion in the sY86 site within the AZFa region, accompanied by partial deletion in the AZFc region, yet displaying normal sperm motility. Therefore, we contend that while YCMs detection is widely applied in screening and diagnosing reproductive disorders, additional extended analyses and genetic testing are essential when partial deletions are detected in the AZF region using STS, to validate and ensure the accuracy of data, thus providing more precise predictions of patients’ spermatogenic status. We hope that our findings will serve as a reference and guide for clinical decision-making and the implementation of assisted reproductive technologies for such patients. The purpose of the study is to validate the necessity of extended analysis in evaluating AZF deficiency patterns by reporting a family in which two males with normal fertility were confirmed to carry sY86 deletion in the AZFa region and partial deletion in the AZFc region.

## 2 Materials and methods

### 2.1 The participant

A 37-year-old man presented to the Reproductive Medicine Center of Sichuan Provincial Maternity and Child Healthcare Hospital for one blighted ovum and one biochemical pregnancy. The patient denied any history of male reproductive system diseases such as prostatitis, orchitis or parotitis, as well as any other systemic illnesses or surgical history. Routine sperm analysis and AZF detection screening were conducted for this patient. Unusual results indicated a normal sperm routine analysis along with partial deletions of AZFa and AZFc areas. To facilitate further genetic counselling, this patient and his father (65 years old) gave informed consents for participation in this study, and the study was approved by Medical Ethics Committee of Sichuan Provincial Maternity and Child Healthcare Hospital.

### 2.2 Semen analysis

Four independent semen routine analyses of different time points were performed for the case according to World Health Organization (WHO) Laboratory Manual for the Examination and Processing of Human Semen (fifth Edition). Volume, pH, sperm concentration and motility, as well as sperm morphology, were recorded. The semen routine analyses were conducted in the Andrology Laboratory of the Sichuan Provincial Maternity and Child Healthcare Hospital.

### 2.3 Evaluation of Y chromosome microdeletion

Analysis was performed using the Y Chromosome Microdeletions Detection Kit (PCR capillary electrophoresis method) (Yaneng Biotechnology Co., Ltd., Shenzhen, China). According to the manufacturer’s protocol, genomic DNA was isolated from peripheral blood lymphocytes. Evaluation of Y chromosome microdeletion was performed for both the case and his father. Multiplex polymerase chain reaction of the following sequence-tagged sites was performed to detect the deletions: AZFa (sY84 and sY86), AZFb (sY127 and sY134), and AZFc (sY254 and sY255). ZFX/ZFY and SRY were used as internal control genes. External positive or negative control DNA was obtained from a man with normal spermatogenesis and a woman with normal fertility.

### 2.4 Male AZF Whole Area Testing Panel

#### 2.4.1 Panel sequencing workflow

Peripheral blood samples were collected from the patient and his father. Genomic DNA was extracted using the QIAamp DNA Blood Mini Kit, followed by agarose gel electrophoresis and Qubit quality control to ensure DNA integrity, concentration, and purity (total mass ≥ 2.5 μg, sample concentration ≥100 ng/μL, OD260/280 between 1.8–2.0, no significant degradation of DNA). The extracted gDNA was randomly fragmented into 250 ∼ 300 bp fragments using Covaris S220, followed by library construction using the NEBNext Ultra II DNA Library Prep Kit (NEB, United States) for Illumina. Following this, the amplified library underwent capture using IDT probes (Integrated DNA Technologies, United States). These probes, individually synthesized and approximately 60-120bp in length, are biotinylated at the 5′end, facilitating the capture of specific genomic regions. The capture process involves distinct steps: specific probe hybridization, binding of magnetic beads to the probes for region-specific capture, removal of unbound probes and nonspecifically bound unstable libraries through washing, and amplification of the captured libraries via PCR, thus achieving targeted region library capture. Subsequently, targeted region capture was performed using IDT probes (Integrated DNA Technologies, United States). Following capture, the target region library was amplified by PCR, and library quantification and fragment length determination were performed using the Qubit dsDNA HS Assay Kit and Agilent2100 Bioanalyzer system (Agilent DNA 1000 Kit), respectively. The captured library was then subjected to cluster generation using the cBot Cluster Generation System (Illumina, United States) with TruSeq PE Cluster Kit V4 reagents, followed by 150 PE sequencing using TruSeq SBS Kit V4-HS reagents and NextSeq 550Dx platform (Illumina, United States).

#### 2.4.2 Sequencing data bioinformatics and genetic variation analysis

Sequencing data were aligned using the BWA-MEM + samtools + picard + GATK process, with the GRCh37/hg19 reference genome. And the comparison results were sorted to remove redundancy and quality correction before conducting variable site sequencing analysis. Samtools and in-house scripts were utilized to calculate sequencing depth, coverage, alignment rate, and duplication rate. SNV/InDel detection was performed using Samtools and GATK, with variant annotation conducted using Annovar software. The content primarily encompasses genetic and regional information, disclosing the positional information of variant loci, gene names, distribution of gene functional regions, and types of variations.

### 2.5 Whole exome sequencing

#### 2.5.1 Sequencing procedure

Genomic DNA was extracted from whole blood samples using the QIAGEN DNA Blood Midi/Mini kit (Qiagen GmbH, Hilden, Germany), and a DNA library was built. The pre-library was subjected to liquid-phase hybridization capture using the NanoWES chip standard procedure. The captured products were then washed, collected, PCR amplified, purified, and resulted in the exome library, which was quantified using qPCR. High-throughput sequencing was performed on the Illumina Novaseq6000 platform (Illumina, San Diego, United States), and raw data were processed using CASAVA v1.82. More than 85% of the data quality bases meet the Q30 or above (≥Q30) standard, and more than 95% of the bases meet the Q20 or above (≥Q20) standard, with a duplication rate not exceeding 30%.

#### 2.5.2 Data analysis

Quality-filtered genomic sequencing data were aligned to the human reference genome (hg19/GRCh37) using Burrows-Wheeler Aligner, and PCR duplicate sequences were removed using Picard v1.57. Variant detection analysis was conducted using the Verita Trekker^®^ variant detection system developed by Berry Gene, in conjunction with GATK. Variant annotation was performed using ANNOVAR (Wang et al., 2010) and the Enliven^®^ variant annotation and interpretation system developed by Berry Gene. Annotation databases included population databases such as gnomAD, the 1000 Genomes Project, Berrybig data, and dbSNP, as well as prediction algorithms like SIFT, FATHMM, MutationAssessor, CADD, and SPIDEX (Xiong et al., 2015). Disease and phenotype databases such as OMIM, ClinVar, HGMD, and HPO were also utilized. Variant interpretation followed the American College of Medical Genetics and Genomics (ACMG) guidelines (Richards et al., 2015), categorizing variants into five classes: “pathogenic,” “likely pathogenic,” “uncertain significance,” “likely benign,” and “benign”. Considering ACMG criteria, pathogenic evidence, clinical summaries of associated diseases, and genetic models, thorough interpretation was performed for variants in exonic regions with a minor allele frequency <0.5% or those affecting splicing.

## 3 Results

### 3.1 Basic clinical features, medical history and semen analysis

The male participants included in this study exhibited no chromosomal abnormalities and tested positive for the SRY gene. They denied any history of male reproductive system diseases. However, they reported experiencing one blighted ovum and one biochemical pregnancy (Table 1). Hormone level tests (Supplementary Table S1) and color ultrasound in gonadal structures (Table 2) showed no significant abnormalities. Subsequently, we analyzed the results of the patient’s semen routine analysis at four different time points. The results indicated that there was an increase in sperm morphological abnormalities, primarily characterized by head abnormalities, with an average head abnormality rate of 98.8%, with no significant abnormalities in other semen parameters (Supplementary Table S2). Sperm chromatin and nuclear protein maturity no significant abnormalities (Supplementary Table S3).

**TABLE 1:** Basic clinical features and medical history of the patient.

Age	SRY^a^	Karyotype	Past medical history	Biochemical pregnancy	Blighted ovum
37	(+)	Normal (46, XY)	Deny	Once	Once

a SRY (Sex-determining Region on the Y Chromosome), (+) means no absence.

**TABLE 2:** Color ultrasound indicated no abnormality in gonadal structures of the Patient.

Testes (mL)		Epididymidis (cm)						Seminal vesicle (cm)		Vas deferens	Ejaculatory duct
Left	Right	Caput		Corpus		Cauda					
		Left	Right	Left	Right	Left	Right	Left	Right		
13.6	17.8	0.6		0.2		0.3		3.1 × 1.4	3.6 × 1.1	(+)^a^	(+)^a^

^a^ (+) means no absence.

### 3.2 Two methods were performed in this study to verify the Y chromosome microdeletions in the family

To exclude the possibility of Y chromosome microdeletions in the patient, we utilized real-time fluorescence quantitative PCR to test six sequence-tagged sites recommended by the European Academy of Andrology and the European Molecular Genetics Quality Network, namely, sY84 and sY86 in the AZFa region, sY127 and sY134 in the AZFb region, and sY254 and sY255 in the AZFc region. As shown in Supplementary Figure S1 and Supplementary Table S4, both the patient and his father exhibited a deletion at the sY86 site, while no deletions were observed at the remaining five sites, including sY84.

**FIGURE 1:**
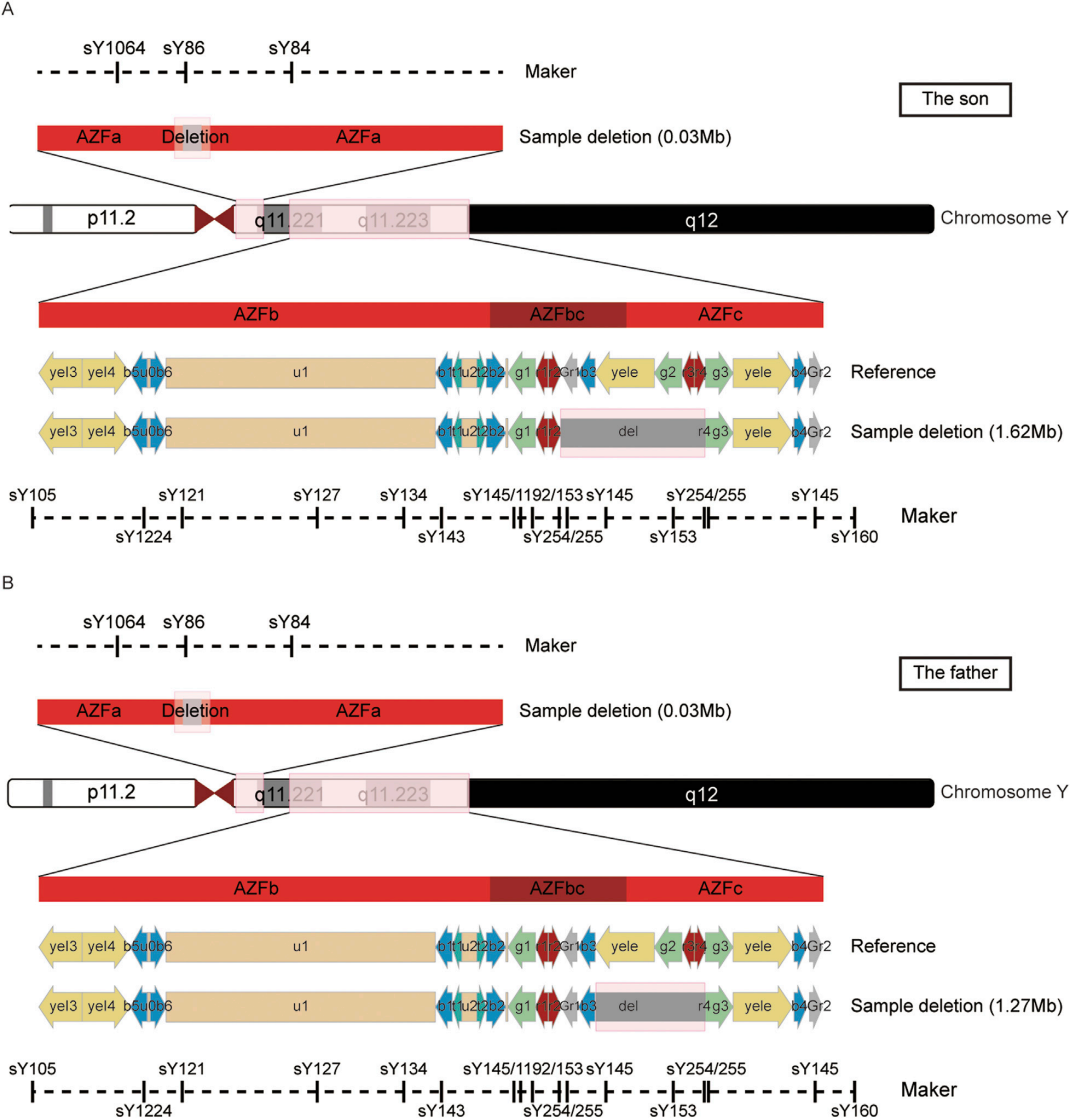
The detailed information of the AZFa and AZFc deletion region in the son **(A)** and father **(B)**.

**FIGURE 2:**
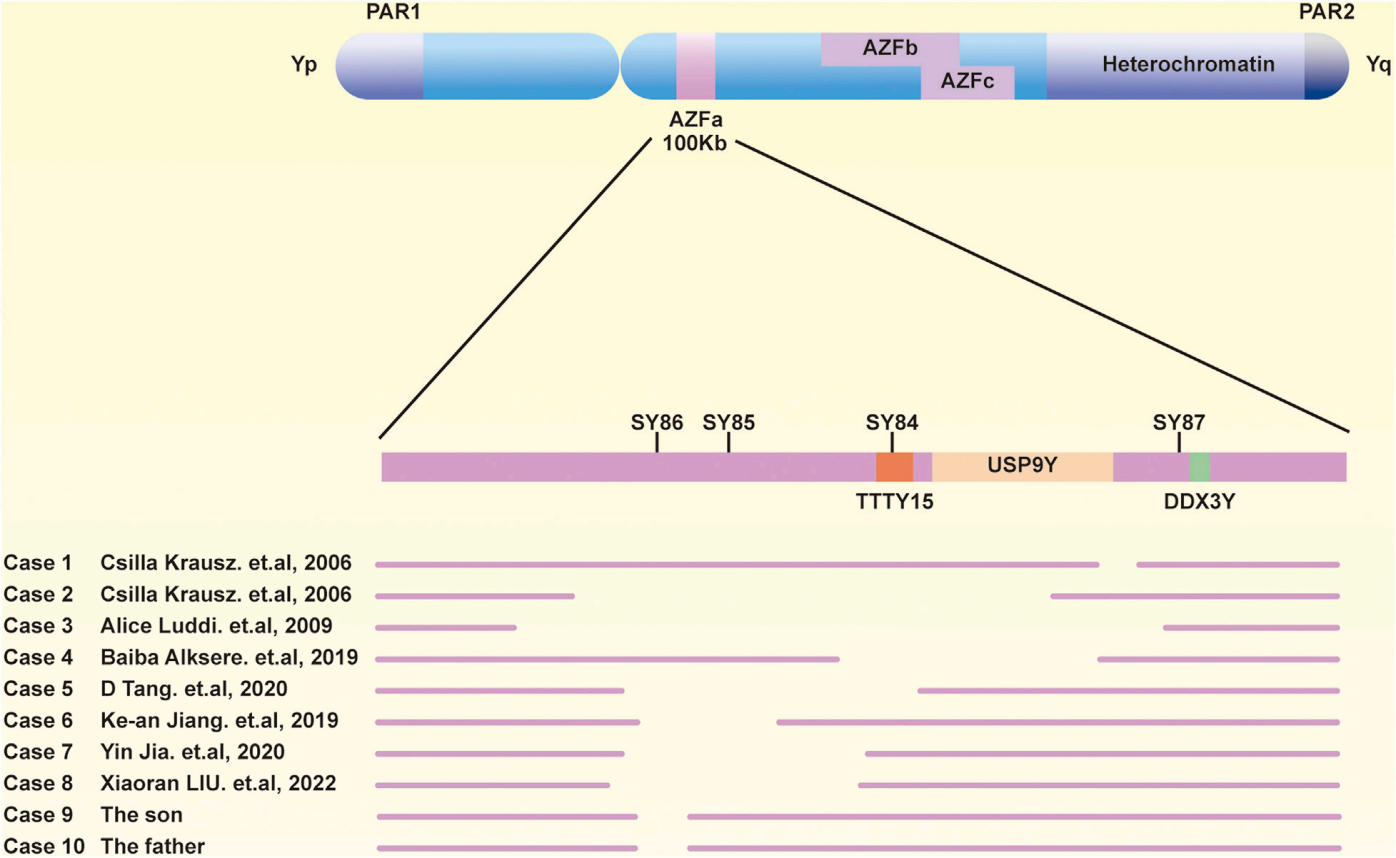
Previous reports and current sections of AZFa are deletions. A schematic diagram of the Y chromosome, including three AZF regions.

To further delineate the specific scope of the AZFa region deletion, we conducted a comprehensive examination of the AZFa, AZFb, and AZFc regions using the Jing Yue Male AZF Whole Area Testing Panel. Additionally, both individuals were detected with a gr/gr deletion in the AZFc region, with the specific deleted region being Yq11.223–11.23 (25,505,378–27,120,665), spanning 1.6 M in the son and Yq11.223–11.23 (25,848,831–27,120,665), spanning 1.3 M in the father (Supplementary Table S5). The graphical representation of the specific deleted regions for the patient and his father is depicted in Figure 1. Furthermore, a summary of the previously reported and the AZFa deletion regions in this case for the father-son pair is illustrated in Figure 2.

### 3.3 Whole exome sequencing

Considering the patient’s nearly 100% abnormal sperm head morphology (98.8%), which has not been definitively linked to Y chromosome microdeletions in previous reports, we further performed whole exome sequencing on the patient’s peripheral blood to screen for potential pathogenic genes associated with sperm head morphology abnormalities. Following the guidelines from the American College of Medical Genetics and Genomics (Richards et al., 2015), and conducting searches in public databases such as HPO, OMIM, and GHR for variants related to clinical phenotypes or diseases, no pathogenic SNVs or InDels clearly associated with the phenotype of this case were detected in the sample. However, two candidate variant sites were identified, as shown in Supplementary Table S6. Among them, the mutations c.12080A > T and c.11176C > T in DNAH9 are variants of uncertain significance.

## 4 Discussion

Y chromosome microdeletions have garnered significant attention as a key etiological factor in male infertility. Among YCMs, deletions within the AZF region are most commonly associated with disruptions in spermatogenesis and male infertility. Currently, the detection of YCMs, particularly AZF deletions, primarily relies on the evaluation of STS. The expert consensus on Y-chromosome microdeletion detection released by the European Society of Andrology in 2012 proposed the detection of six STS loci for AZF gene microdeletions. In the 2015 Consensus of Male Reproductive Genetics Examination Experts released by the Andrology Branch of the Chinese Medical Association, the Y-chromosome AZF microdeletion detection sites were expanded to 8, and the detection of AZFc region sY145 and sY152 sites was added (Shang et al., 2015).

YCMs represent a significant cause of severe oligozoospermia and azoospermia, with deletion frequencies ranging from 2% to 11.5% among infertile males (Simoni et al., 2008; Krausz et al., 2014). Complete deletion of the AZFa region can lead to azoospermia, with the patient’s testicular tissue pathology type being SCOS(Colaco and Modi, 2018). Partial deletion of the AZFa region, however, is relatively rare with complex clinical phenotypes, ranging from completely normal to azoospermia (Wei et al., 2015). Although partial deletion of AZFa is considered to exhibit a milder phenotype than complete deletion, only a few cases have been reported to have normal sperm parameters and fertility (Tang et al., 2020) (Table 3).

**TABLE 3:** Partial absence of AZFa with normal sperm parameters and fertility.

AZFa deletion site	Fertility	Clinical symptoms	Specific deletion fragment	Authors and references
sY86	unexplained infertility	clinical examination, semen analysis and chromosome analysis showed no abnormalities	-	Jiang et al. (2019)
sY83, sY1064, sY86	normal fertility	clinical examination, semen analysis and chromosome analysis showed no abnormalities	son and father: chrY:14,415,452–14,623,795,208 kb	Jia et al. (2020)
sY83, sY1064, sY86	normal fertility	clinical examination, semen analysis and chromosome analysis showed no abnormalities	son and father: Yq11.21 (14,416,038–14,622,345), 206.3 kb	Xiaoran et al. (2022)
sY84, sY1323	normal fertility	clinical examination, semen analysis and chromosome analysis showed no abnormalities	son and father: 800 kb of AZFa region deletions, *USP9Y* (5‘end deletion), no effect on *DDX3Y*	Alksere et al. (2019)
sY84, sY86	normal fertility	semen analysis showed no abnormalities	son and father: chrY: 12,470,437–12,690,385, 215kb, no effect on *USP9Y* and *DDX3Y*	Tang et al. (2020)

The AZFa region spans approximately 1,100 kb and encompasses genes closely associated with spermatogenesis, notably *USP9Y* and *DDX3Y*. USP9Y may play a marginal role in spermatogenesis (Sun et al., 1999; Krausz et al., 2006; Ferlin et al., 2007; Luddi et al., 2009; Alksere et al., 2019), and consistent with this hypothesis is the inactivation of homologous genes in chimpanzees and bonobos. Compared with the USP9Y gene, DDX3Y plays a more critical role in spermatogenesis and is considered to be the main spermatogenic gene in the AZFa region, involved in the differentiation regulation of spermatogonia and spermatocytes. Its absence in the AZFa region can lead to severe oligozoospermia or azoospermia. Currently, there are no reported cases of its absence with normal semen (Kleiman et al., 2012). Our study is similar to that of Ke an Jiang et al. where individual sY86 site deletions do not seem to affect patient semen parameters. This may be because the deletion range does not involve the USP9Y and DDX3Y coding genes in the AZFa region. This also suggests that in clinical practice, the detection of sY86 site deletion by the six point method alone does not represent a complete deletion of the AZFa region. Even if both sY84 and sY86 site deletions are detected simultaneously, it only indicates a high possibility of complete deletion of the AZFa region. However, more detailed research should be conducted on the AZFa region. Patients with partial deletion of sY84 and sY86 sites may have normal semen parameters. According to the latest guidelines, molecular detection in the AZFa region currently uses two STS markers: sY84 and sY86. Both are located upstream of the *USP9Y* and *DDX3Y* genes. According to the pathogenic mechanism of the deletion and existing data, when sY84 and sY86 are simultaneously deleted, not 100% of AZFa is completely deleted, but the probability is very high. It is possible (although rare) that both loci are missing without affecting the two genes of AZFa, or only the *USP9Y* gene is affected. Therefore, AZFa deletion detection requires mandatory extended deletion analysis, including proximal boundary sY82, *etc.* Based on our research findings, we suggest modifying the guidelines for detecting deletions in the AZFa region to be more selective towards the DDX3Y gene and exclude the potentially benign USP9Y gene microdeletion alone. However, non-obstructive azoospermia patients with concurrent absence of sY84 and sY86 typically do not undergo microTESE, thus there was no relevant data on adverse outcomes resulting from the lack of extended analysis at present. Recent data indicates that in a considerable number of AZFa completely missing cases, the proximal breakpoint marker sY83 (previously used as an equivalent option for sY1064) still exists. Therefore, in order to avoid misdiagnosing complete AZFa deficiency as partial deficiency, it is strongly recommended to use sY1064 instead of sY83 in extended deficiency analysis (or at least sY1064 should be tested to confirm that it is indeed complete deficiency in sY83 positive cases). These two AZFa genes are located further away from the AZFa region, so detecting the recommended distant AZFa site is highly correlated with the prognosis of TESE, and the exact location of the proximal breakpoint (whether sY83 is present or not) does not actually affect the clinical interpretation of the deletion. When encountering patients with azoospermia or oligoasthenozoospermia who only have partial AZFa region deletions, it is not enough to only perform Y chromosome microdeletion testing. Other genetic or non genetic factors may play a more important role in phenotype development. If the AZFa region is not completely missing, more detailed and extensive analysis is needed, such as screening for other genetic markers, conducting whole exome testing to analyze for male infertility pathogenic mutations, and conducting testicular biopsy and genetic counseling. Complete deletion of AZFa leads to SCOS and azoospermia, whereas partial deletions are believed to manifest with milder phenotypes compared to complete deletions, albeit only a handful of cases have been reported with normal sperm parameters and fertility. According to reports, deletion of the UTY gene in the AZFa region may result in the complete absence of male germ cells, and that UTY is also of GBY the overlapping gonadoblastoma susceptibility Y region (Vogt et al., 1996). These studies suggest that phenotypic differences in spermatogenesis abnormalities caused by ACF deletions may be related to the affected functional genes. However, there is currently no definitive literature reporting a correlation between AZF deletions and sperm head malformations. Therefore, when sperm head malformations, a type of sperm abnormality not clearly reported to be associated with AZF deletions, are identified in patients, whole-exome sequencing represents a potential means to further explore the underlying disease mechanisms. Notably, the same deletion type can exhibit varying clinical phenotypes due to differences in genetic backgrounds such as ethnicity and geography. Consequently, the relationship between rare microdeletions on the Y chromosome and spermatogenesis necessitates substantiation through extensive clinical data. Additionally, given that semen parameters may appear normal in these patients, such atypical deletions are obligatorily transmitted to male offspring, and thus, patients should be informed of the risk of producing male offspring with impaired spermatogenesis.

Phenotypic variability is observed in individuals with complete AZFc deletions, ranging from normal sperm parameters to mild oligozoospermia and even azoospermia. About two-thirds of patients exhibit azoospermia, with approximately 50% of them being able to retrieve sperm through testicular puncture, while the remaining one-third typically presents with oligozoospermia. Therefore, theoretically, patients with AZFc region deficiency can obtain their own offspring through intracytoplasmic sperm injection (ICSI) technology. Other types of deletions (such as b1/b3, b2/b3, gr/gr, *etc.*) belong to partial deletions, Repping et al. (2003) first reported the association between the gr/gr deletion and spermatogenesis impairment in 2003, although subsequent studies have yielded inconsistent results (Table 4). DAZ1/DAZ2 deficiency is associated with male reproductive dysfunction and may be a risk factor for male infertility. Among them, the DAZ gene encodes an RNA binding protein and is considered a major candidate gene that may be related to male infertility (Saxena et al., 2000). The AZFc region contains two sets of DAZ gene copies (DAZ1/DAZ2 and DAZ3/DAZ4), totaling four DAZ gene copies. Different gr/gr subtypes exhibit varying patterns of DAZ gene copy deletions. Association analysis shows that the correlation between gr/gr and idiopathic infertility is only found in gr/gr deletion subtypes that have lost copies of the DAZ1/DAZ2 gene (Giachini et al., 2008; Stouffs et al., 2008; Yang et al., 2008). In our study, this father son pair not only had partial deletions in the AZFa region, but also had partial deletions in the AZFc region. The missing region of the son is gr/gr (r2/r4) (1.62 Mb), while the father is gr/gr (g3/r4) (1.3 Mb). The missing region does not include copies of the DAZ1/DAZ2 gene, which may be the reason why significant abnormalities in sperm were not observed in this father son pair. Therefore, partial deletions in the AZFc region, such as b1/b3, b2/b3, and gr/gr, are currently considered risk factors for reduced sperm count and are significantly associated with genetic backgrounds. The risk of offspring developing complete deletions increases, as evident from the larger deletion region in the son compared to the father, underscoring the importance of early fertility assessments in male descendants.

**TABLE 4:** The association between partial deletion of AZFc and spermatogenesis impairment.

Participant	Study population	Deletion rate	Conclusion	Authors and references
Infertile men (634), normozoospermic fertile men (248)	Han-Chinese from eastern China	Patient: gr/gr (7.0%), b2/b3 (8.9%) Control: gr/gr (7.7%), b2/b3 (3.2%)	the absence of gr/gr, b2/b3 is not significantly correlated with the occurrence of oligospermia, only with different distribution frequencies in different populations	Wu et al. (2007)
Patients with spermatogenic impairment (296) and healthy controls (280)	Han Chinese from East China	Patient: gr/gr (8.1%), b2/b3 (8.8%) Control: gr/gr (7.1%), b2/b3 (6.4%)		Zhang et al. (2007)
Infertile males with various spermatogenic impairments (121), healthy donors (95)	Chinese	Patient: gr/gr (12.40%), b2/b3 (4.96%) Control: gr/gr (13.68%), b2/b3 (1.05%)	demonstrated associations between AZFc region gr/gr and b2/b3 deletions and male spermatogenesis disorders	Wang et al. (2016)

Furthermore, despite normal semen parameters observed in the patient’s four semen analyses at different time points, there was a notable increase in abnormal sperm morphology, predominantly characterized by head abnormalities, with an average head abnormality rate of 98.8%. Considering genetic factors, especially key gene mutations in sperm head development, which may lead to abnormal sperm head morphology, we further performed whole exome sequencing on this patient and analyzed the sequencing data. According to the ACMG guidelines (Richards et al., 2015), as well as screening for phenotypic or disease-related gene variations in public databases such as HPO, OMIM and GHR, no pathogenic SNV or InDel variants related to the phenotype of this case were detected in the sample of the test subjects, but the presence of other unreported or unidentified mutations cannot be ruled out. Given the limited data available from only a father-son pair within a single pedigree, the absence of extensive statistical support may compromise the reliability of the conclusions. Therefore, further research with a larger sample size is necessary to validate these findings and ensure their generality.

## Data Availability

The original contributions presented in the study are publicly available. This data can be found here: https://www.ncbi.nlm.nih.gov/sra/PRJNA1202094.
