# Supplementary material for: A family with normal sperm motility carrying a sY86 deletion in AZFa region and partial deletion in AZFc region
Source: Front Genet. 2025 Jan 9;15:1519774. doi: 10.3389/fgene.2024.1519774 (PMC11754199; doi:10.3389/fgene.2024.1519774)
Supplement: Supplementary file 1 [file Table1.docx]

**URLs of these databases**

Picard v1.57 (http://picard.sourceforge.net/)

GATK (https://software.broadinstitute.org/gatk/)

gnomAD (http://gnomad.broadinstitute.org/)

the 1000 Genomes Project (http://browser.1000genomes.org)

dbSNP (http://www.ncbi.nlm.nih.gov/snp),

SIFT (http://sift.jcvi.org),

FATHMM (http://fathmm.biocompute.org.uk),

MutationAssessor (http://mutationassessor.org),

CADD (http://cadd.gs.washington.edu),

OMIM (http://www.omim.org),

ClinVar (http://www.ncbi.nlm.nih.gov/clinvar),

HGMD (http://www.hgmd.org),

HPO (https://hpo.jax.org/app/)

**Table S1. Sex hormone levels**

| **Sex hormone** | **Concentration** | **Unit** | **Reference value** |
| --- | --- | --- | --- |
| Estradiol (E2) | 30.0 | Pg/ml | 0-39.8 |
| Progesterone (P) | 0.53 | ng/ml | 0.28-1.22 |
| Testosterone (T) | 4.51 | ng/ml | 1.97-6.70 |
| Luteinizing hormone (LH) | 4.0 | IU/L | 1.5-9.3 |
| Follicle stimulating hormone (FSH) | 6.1 | IU/L | 1.4-18.1 |
| Prolactin (PRL) | 5.6 | ng/ml | 2.1-17.7 |
| Dehydroepiandrosterone sulfate (DHEAS) | 228 | μg/dl | 80-560 |
| Androstenedione (AND) | 1.92 | ng/ml | 0.6-3.1 |
| Sex hormone binding globulin (SHBG) | 30.2 | nmol/L | 11.54-54.49 |
| Free androgen index (FAI) | 51.8 |  | 26.18-107.07 |

a Sex-determining region Y (SRY): (+) means detectable.

**Table S2. Several routine semen analysis of the patients at different times**

| **Project** | **Result 1** | **Result 2** | **Result 3** | **Result 4** | **Units** | **Reference value** |
| --- | --- | --- | --- | --- | --- | --- |
| Color | Pale | Pale | Pale | Pale |  |  |
| Viscosity | Normal | Normal | Normal | Normal |  |  |
| Volume | 5.4 | 4.5 | 4.1 | 4.0 | ml | >=1.5 |
| Liquefaction time | 30 | 30 | 30 | 30 | min | <=60 |
| pH | 7.4 | 7.4 | 7.5 | 7.5 |  | >=7.2 |
| Sperm concentration | 26.0 | 23.9 | 17.8 | 21.1 | 10^6^/ml | >=15 |
| Total sperm count | 140.4 | 107.6 | 73 | 84.4 | 10^6^/one ejaculation | >=39 |
| Viability | 55 | 92 | 80 | 58 | % | >=58 |
| Antisperm antibody  (MAR method) | negative | negative | negative | negative |  | negative |
| Round cell | 0.4 | 0.3 | 0.6 | 0.3 | 10^6^/ml | Leukocyte<1 |
| Total motility | 40 | 80 | 53 | 53 | % | >=40 |
| Progressive motility | 36 | 78 | 46 | 50 |  | >=32 |
| Sperm count for morphology analysis | 218 | 209 | 209 | 206 |  |  |
| Sperm count  of normal morphology | 2 | 1 | 5 | 1 |  |  |
| Percentage of normal morphology | 0.9 | 0.5 | 2.4 | 0.5 | % | >=4 |
| Sperm count with defects | 216 | 208 | 204 | 205 |  |  |
| Sperm count with  head defects | 216 | 208 | 204 | 204 |  |  |
| Sperm count with neck and middle piece defects | 10 | 18 | 13 | 20 |  |  |
| Sperm count with tail defects | 20 | 11 | 17 | 29 |  |  |
| Total number of defects | 246 | 237 | 234 | 253 |  |  |
| Teratozoo-  spermia index (TZI) | 1.14 | 1.14 | 1.15 | 1.23 |  |  |
| Sperm deformity index(SDI) | 1.13 | 1.13 | 1.12 | 1.23 |  |  |

**Table S3. DNA Fragmentation rate and nucleoprotein maturity**

| **Project** | **Result 1** | **Result 2** | **Result 3** | **Result 4** | **Result 5** | **Units** | **Reference value** |
| --- | --- | --- | --- | --- | --- | --- | --- |
| DNA fragmentation index (DFI) | 35.2 | 22.1 | 27.3 | 20.6 | 18.1 | % | <30% |
| High DNA staining (HDS) | 7.7 | 11 | 9.3 | 11.4 | 7.8 | % | ≤15% |
| Percentage of sperm with mature nuclear protein | 83.0 | 79 | 87 | 90 | 73 | % | ≥70% |

**Table S4. Interpretation of the classical six STS sites**

|  | **Component A** | | | | **Component B** | | | | **Results** |
| --- | --- | --- | --- | --- | --- | --- | --- | --- | --- |
| **Fluorescence Channel** | **ROX** | **VIC** | **FAM** | **CY5** | **ROX** | **VIC** | **FAM** | **CY5** |  |
| **STS site** | **SY84** | **SY127** | **SY255** | **ZFX/Y** | **SY86** | **SY134** | **SY254** | **SRY** |  |
| **Fluorescent channel amplification detection** | **+** | **+** | **+** | **+** | **+** | **+** | **+** | **+** | Normal  male |
|  | **-** | **+** | **+** | **+** | **-** | **+** | **+** | **+** | AZFa  deletion |
|  | **+** | **-** | **+** | **+** | **+** | **-** | **+** | **+** | AZFb  deletion |
|  | **+** | **+** | **-** | **+** | **+** | **+** | **-** | **+** | AZFc  deletion |
|  | **+** | **-** | **-** | **+** | **+** | **-** | **-** | **+** | AZFb+c deletion |
|  | **-** | **-** | **-** | **+** | **-** | **-** | **-** | **+** | AZFa+b+c deletion |
|  | **-** | **-** | **-** | **+** | **-** | **-** | **-** | **-** | Normal  female |
| **Son** | **+** | **+** | **+** | **+** | **-** | **+** | **+** | **+** | Partial  AZFa  deletion |
| **Father** | **+** | **+** | **+** | **+** | **-** | **+** | **+** | **+** | Partial  AZFa  deletion |

**Table S5. Patial AZFa and AZFc deletion were identified by NGS**

|  | **Detected**  **Variant** | **Chromosome** | **Start position** | **End position** | **Variant**  **type** | **Variant length** | **Genes included** | **Missing type** |
| --- | --- | --- | --- | --- | --- | --- | --- | --- |
| **Son** | Seq [GRCh37]  del(Y)(q11.21) | chrY | 1460  7372 | 1463  7973 | Hemizygote  deletion | 30.6kb | NA | Patial  AZFa  deletion |
|  | Seq[GRCh37]  del(Y)(q11.223q11.23) | chrY | 2550  5378 | 2712  0665 | Hemizygote  deletion | 1.6M | TTTY3B, TTTY4B, DAZ4, etc | Patial  AZFc  deletion |
| **Father** | Seq[GRCh37]  del(Y)(q11.21) | chrY | 1460  7372 | 1463  7973 | Hemizygote  deletion | 30.6kb | NA | Patial  AZFa  deletion |
|  | Seq[GRCh37]  del(Y)(q11.223q11.23) | chrY | 2584  8831 | 2712  0665 | Hemizygote  deletion | 1.3M | TTTY3B, TTTY4B, DAZ4, etc | Patial  AZFc  deletion |

**Table S6. Variants related to phenotype but with insufficient evidence to prove pathogenicity**

| **Gene**  **name** | **Variant**  **position** | **Gene**  **subregion** | **HGVS** | **Variant Type** | **Heterozygosity** | **Variant rating** | **Diseases and**  **genetic patterns** |
| --- | --- | --- | --- | --- | --- | --- | --- |
| *DNAH9* | chr 17: 11930068-  11930068 | exon63 | NM_001372.4  : c.12080A>T:  p.H4027L | missense_variant | Heterozygous | VUS | Primary ciliary dyskinesia type 40, AR |
| *DNAH9* | chr17:  11891840-  11891840 | exon58 | NM_001372.4  : c.11176C>T:  p.R3726W | missense_variant | Heterozygous | VUS |  |

The reference database version is: human Genome 38 (hg38/GRCh38)

VUS: Variant of Unknown Significance

AD: Autosomal dominant

AR: Autosomal recessive


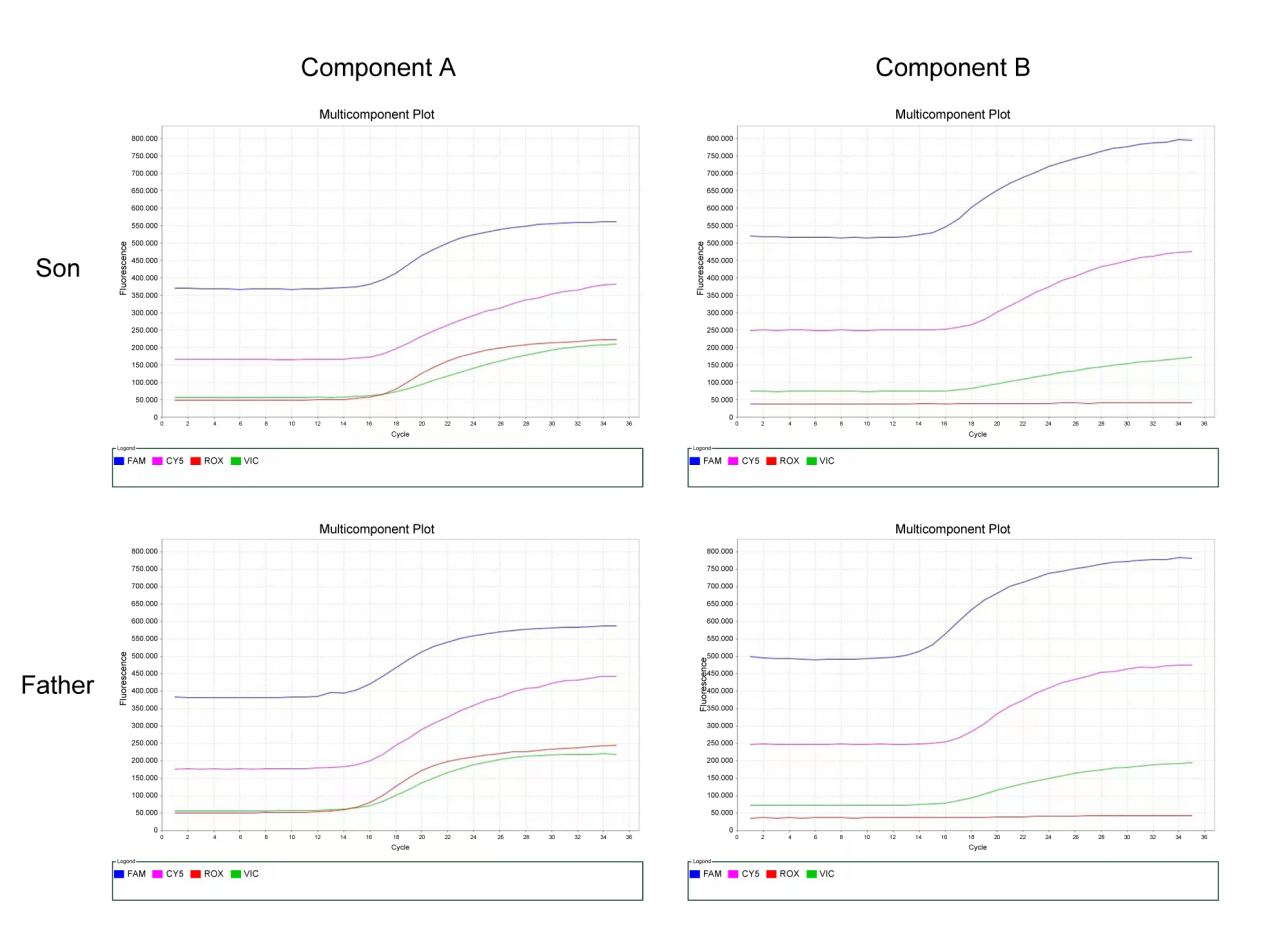


**Figure S1. The detection of the six STS sites indicated the absence of SY86 in the son and the father**
